# Physicians’ Self-Perceived Competence on Breaking Bad News to Parents of Children with Neurodisabilities

**DOI:** 10.3390/children10121854

**Published:** 2023-11-26

**Authors:** Ophélie Fiorellino, Christopher John Newman

**Affiliations:** 1 Faculty of Science and Medicine, Medicine Section, University of Fribourg, 1700 Fribourg, Switzerland; ophelie.aubert@rhne.ch; 2 Pediatric Neurology and Neurorehabilitation Unit, Woman Mother Child Department, Lausanne University Hospital, University of Lausanne, 1011 Lausanne, Switzerland

**Keywords:** child, disability, breaking bad news, physician experience

## Abstract

Delivering difficult news to parents of children with neurodisabilities, often involving new diagnoses, prognosis changes, or declines in function or health, presents a complex task. Our aim was to assess physicians’ self-perceived competence in breaking bad news (BBN) within this context. An online survey was administered to neuropediatricians and developmental and rehabilitation pediatricians in Switzerland. Among 247 invited physicians, 62 (25.1%) responded (age of 51 ± 11 years; M/F ratio of 2:3). They rated their BBN competence at 7.5 ± 1.6 out of 10. Factors significantly associated with self-perceived competence in uni- and multivariate analyses included years of professional experience (≤10 years: 6.2 ± 1.8; >10 years: 8.2 ± 0.8), and region of pregraduate training (Switzerland: 7.3 ± 1.6; European Union: 8.3 ± 0.9). The respondents highlighted the positive roles of professional and personal experience, quality relationships with families, and empathy in BBN. In summary, physicians generally expressed a sense of competence in delivering difficult news to parents of children with neurodisabilities. They underscored the significance of life experiences and certain individual qualities in their effectiveness. These findings provide valuable insights into enhancing professional training and support in this crucial yet underexplored aspect of medical practice.

## 1. Introduction

Delivering difficult news to parents of children with neurodisabilities is a complex and emotionally charged endeavor [1]. Neurodisabilities refer to a group of enduring conditions, whether congenital or acquired, arising from impairments in the brain and/or neuromuscular system, and leading to functional restrictions [2]. These conditions may not always have a specific diagnostic label and can exhibit varying characteristics, both in isolation or combination, with a wide spectrum of severity and complexity. They can manifest as challenges in aspects like mobility, cognitive function, sensory perception (including hearing and vision), communication, emotional well-being, and behavioral patterns [3]. Delivering news about these children involves communicating information that has the potential to profoundly reshape parents’ understanding of their child’s future. Such information may encompass a new diagnosis, a shift in prognosis, or a decline in their child’s functional abilities or overall health. This research investigates the issue of breaking bad news (BBN) within the context of healthcare providers who specialize in caring for children with neurodisabilities, namely neuropediatricians, developmental pediatricians, and rehabilitation pediatricians.

Acknowledging the unique challenges faced by physicians caring for children with neurodisabilities and recognizing the delicate nature of these discussions, the impact of delivering such news extends beyond the medical diagnosis itself. It ripples through the emotional landscape of families, influencing their perceptions, decisions, and ulterior experiences with the healthcare system. Most studies on BBN in childhood neurodisabilities have focused on parent experiences [1,4,5,6,7,8,9,10,11,12,13,14]. Overall, these studies underlined the importance of timely communication when delivering bad news about a child’s neurological disorder or disability. This included parents being told early and receiving information promptly. Effective communication skills, such as providing detailed information and being direct, were consistently associated with higher parental satisfaction with the disclosure process. Understanding and addressing parental concerns played a pivotal role in enhancing satisfaction [7,14]. This involved acknowledging both the challenges and strengths associated with the child’s condition [9].

The effect of delivering bad news is not confined to patients and their families; it also extends to the healthcare providers themselves [15]. Without proper training, breaking bad news can lead to negative consequences for patients, families, and physicians [16]. Physicians may be fearful of the patient’s or family’s reaction to the news or be uncertain about how to deal with an intense emotional response. Also, the pace of clinical activity may lead a physician to BBN with little forewarning or when other responsibilities are competing for the physician’s attention, and bad news may be delivered in settings that are not conducive to such intimate conversations [17]. Communication skills, empathy, and cultural competence play pivotal roles in shaping the delivery of bad news [18,19,20]. Broadly, studies on teaching strategies to enhance communication skills among physicians indicate training programs can be effective if they span at least one day, prioritize learner-centered approaches, and emphasize skill practice. The most successful training strategies incorporated within these programs encompass role play, constructive feedback, and small group discussions [21]. However, when it comes to medical students, the effectiveness of simulated patients (SPs) in undergraduate BBN education remains uncertain, despite their widespread use. Despite the varied ways SPs have been employed, the advantages of different approaches and the optimal timing and methods of delivery remain unclear [22].

Only two studies have explored physician experiences with BBN within the context of childhood neurodisabilities, each including interviews with around 25 pediatricians [23,24]. Both studies highlighted the shortcomings and challenges in pediatricians’ disclosure practices related to diagnoses in children with neurodisabilities. While experience and attitudes played a role, there were also significant influences from factors such as parents’ emotional state [23], time constraints [24], and the need for improved training and policies [24] to enhance disclosure practices. The anxiety-provoking nature of BBN discussions can evoke emotional responses in physicians [12]. Conversely, when doctors feel competent in conveying difficult news, they can experience a sense of fulfillment, irrespective of the prognosis [25]. Enhancing the preparedness of healthcare professionals for such challenging encounters has been shown to reduce the stress associated with BBN [26] and improve physician comfort and skills [27] and could, therefore, significantly improve their daily practice. It can mitigate emotional detachment that may arise when dealing with difficult reactions from patients and alleviate the emotional fatigue that contributes to physician burnout [28].

Our study endeavors to shed light on the confidence levels and attitudes of healthcare professionals who navigate these intricacies. Specifically, we aimed to explore how these physicians perceive their competence in the task of BBN to identify personal factors associated with self-perceived competence, as well as their beliefs associated with this fundamental aspect of patient care.

## 2. Materials and Methods

### 2.1. Study Design

We conducted a cross-sectional descriptive study via a web-based survey. Because this study included healthcare professionals and was strictly anonymous, the formal agreement by the regional ethics commission was waived, according to Swiss law on research on human beings [29].

### 2.2. Selection Criteria

We approached physicians we considered potentially active in BBN about children with neurodisabilities to participate. These included neuropediatricians, developmental pediatricians, and rehabilitation pediatricians in independent and/or hospital-based practices who were active in Switzerland.

### 2.3. Questionnaire

The survey questionnaire was developed from the inductive thematic analysis of five individual semi-structured interviews designed to explore key aspects of BBN processes in the context of childhood neurological disabilities. We interviewed five physicians specializing in neuropediatrics (*n* = 4) or pediatric rehabilitation medicine (*n* = 1) from the French-speaking area of Switzerland, of whom three were in independent practice and two worked in a hospital; three were women, and two were men, and each had at least 15 years of experience in their specialty. These physicians formed a convenience panel aiming to explore a variety of experiences in BBN (in terms of age, gender, setting, and specialty) and were identified by the second author based on his knowledge of their experience in BBN in the field of childhood neurodisability. The interviews were recorded, transcribed, and coded by the first author using NVivo 14 (Lumivero, Denver, CO, USA). The coding was reviewed and adjusted by the second author. Overarching topics that emerged from the thematic analysis were arranged by importance of professional proficiency to BBN, personal qualities relating to BBN, the role of training, the role of experience, physician relationships with families, and physician emotions associated with BBN. The questionnaires were developed in French and forward translated into German by a bilingual native German-speaking physician and retro-translated into French by a bilingual native French-speaking physician, neither of whom participated in this study. This was to ensure the equivalence of both versions before distribution. The questionnaires were reviewed by two senior physicians specializing in pediatric rehabilitation and neurology for content validation before distribution.

The first section of the questionnaire explored the personal (age, gender) and professional (specialty, duration of professional activity in specialty, region of pregraduate training, and formal training in BBN) characteristics of the participants. The second section measured the participants’ experience and self-perceived competence (on a numerical scale of 1 to 10) in BBN. The third section explored participants’ general views on BBN in their setting by expressing their agreement on a four-point Likert scale (completely disagree, disagree, agree, and completely agree) to statements about the acquired/innate nature of BBN proficiency, the role of professional experience in BBN, the role of personal experience in BBN, the importance of the relationship quality with parents in BBN, and the role of formal training in BBN. Factors potentially associated with certain aspects of these views, as well as with the emotional impact of BBN on the participants, were assessed on a numerical scale of 1 to 10. We instructed participants to respond to all the questions. The questionnaire script is available as a supplementary file (Table S1).

### 2.4. Recruitment

An invitation e-mail was sent to the Swiss Society of Pediatric Neurology, the Swiss Society of Developmental Pediatrics, and the Swiss Academy of Childhood Disability in March 2021 with a request for distribution to our target participants and for feedback on the number of potential participants to determine the participation rate. This e-mail included a link to a web-based survey (SurveyMonkey^®^, San Mateo, CA, USA) and informed the participants that by completing the questionnaire they consented to the use of their anonymous data for the purpose of this study.

### 2.5. Sample Characteristics

A total of 247 physicians were invited via the three professional societies. Sixty-two (25.1%) responded. Their ages were 51.1 ± 10.9 years with a male/female gender ratio of 2:3. Their median professional experience since obtaining their licensure was 15 years (IQR 9.8–25 years). Further participant characteristics are described in Table 1.

Fifty-four questionnaires were fully completed, and the completion rate for all questionnaires was 98.7% (8 questionnaires with between 1 and 3 out of 21 responses missing).

### 2.6. Statistics

Responses were reported by descriptive statistics with means and standard deviations for normally distributed continuous values, medians and interquartile ranges for non-normally distributed continuous values, and proportions and frequencies for categorical values. Distribution of continuous data was assessed with the Kolmogorov–Smirnov test of normality. We reduced the 4-level Likert scale to two categories for analyses (agree, disagree). Univariate associations with self-perceived competence were explored using Mann–Whitney U tests and multivariate associations via linear regression. We did not use statistical methods to infer missing data. *p*-values < 0.05 were considered significant. We used IBM SPSS^®^ Statistics version 27 (IBM, Armonk, NY, USA) to perform the analyses.

## 3. Results

### 3.1. BBN Experience and Self-Perceived Competence

Physicians delivered bad news a median of 18 (IQR: 10–25) times per year. They rated their self-perceived competence at 7.5 ± 1.6 out of a maximal score of 10.

In univariate analyses, self-perceived competence was significantly associated with professional experience (≤10 years: 6.2 ± 1.8; >10 years: 8.2 ± 0.8; *p* < 0.001), gender (M: 8.3 ± 0.8; F: 7.1 ± 1.7; *p* < 0.001), and region of pregraduate training (Switzerland: 7.3 ± 1.6; EU: 8.3 ± 0.9; *p* = 0.013). There were no significant associations with the respondents’ specialty, geographical area of work, frequency of BBN, or formal training in BBN. In the multivariate linear regression analysis, including all aforementioned factors, only professional experience (*p* = 0.002) and region of pregraduate training (*p* = 0.016) remained significantly associated with self-perceived competence.

### 3.2. Physicians Views on BBN

**Figure 1:**
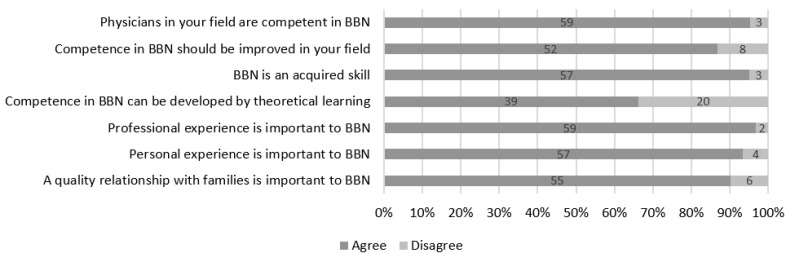
Physician considerations on BBN proficiency.

Overall, a large majority of respondents found their colleagues competent in BBN, that BBN was a point of improvement in their field, and that both professional and personal experience were important to BBN as well as a quality relationship with families. A third of respondents did not agree that theoretical learning could contribute to developing competence in BBN (Figure 1).

Respondents rated the following personal features out of 10 as contributing to BBN in decreasing order of importance: empathy at 9.5 ± 0.8, preparation at 8.9 ± 1.6, adaptability at 7.9 ± 1.9, and personality at 7.8 ± 1.9.

Personal experiences that were rated as contributive to BBN were in decreasing order of importance: personal relationships with people with disabilities as 7.5 ± 1.9, being a parent as 6.5 ± 2.6, experience with grieving as 6.4 ± 2.3, having received bad news as a relative as 6.1 ± 2.5, and having received bad news as a patient as 6.0 ± 2.5.

Factors that contributed to making BBN emotionally impactful for the respondents were in decreasing order of importance: the severity of the child’s diagnosis as 8.5 ± 1.7, the emotional reaction of the child’s parents as 8.1 ± 1.8, and the age of the child as 5.0 ± 2.7. Respondents associated disability severity with the disability’s impact on those close to the child as 8.7 ± 1.3, the disability’s effect on the child’s quality of life as 8.6 ± 1.4, the lack of possible treatments as 8.5 ± 1.8, the disability’s effect of the child’s independence as 8.4 ± 1.5, and the disability’s effect on the child’s life expectancy as 7.4 ± 2.8. There was a large consensus on the usefulness of debriefing after BBN (97% agreement) to improve practice.

## 4. Discussion

Our study found that physicians specializing in the care of children with neurodisabilities have an average self-perceived competence score of 7.5 out of 10, which is relatively high. This suggests that, in general, these physicians feel confident in their ability to communicate difficult information to parents. However, this study also highlighted the role of professional experience and pregraduate training region in influencing self-perceived competence. This finding aligns with previous research emphasizing the importance of both experiential learning [30] and cultural context [31] in shaping physicians’ communication skills.

Professional experience played a significant role in shaping physicians’ self-perceived competence in BBN. More experienced physicians rated themselves as more competent in delivering bad news. This association could be attributed to the accumulation of clinical knowledge, communication skills [32], and a deeper understanding of the nuances involved in delivering difficult information over years of practice [33]. Seasoned physicians often have more exposure to challenging clinical interactions, which may contribute to their confidence in handling BBN discussions. This finding underscores the importance of ongoing professional development, mentoring, and experiential learning to enhance the competence of less experienced physicians.

In the univariate analysis, we found that male physicians had a higher self-perceived competence in BBN compared to their female counterparts, a trend that was previously reported among pediatric specialist registrars [34]. Women physicians have been reported to tend to judge their own skills as lower than men despite similar objective performance [35]. Self-assessment is subject to inaccuracy compared to effective performance for several reasons, including the scoring of idealized rather than actual performance, the scoring of effort rather than achievement, self-deception, or, on the contrary, self-depreciation [36]. Educational, social, and cultural factors certainly act on the gender-observed differences in self-judgment. However, this gender difference was not significant in the multivariate analysis, suggesting that gender alone is not a significant predictor of competence when accounting for other variables. The univariate association was confounded by the fact that there were more male physicians among the more experienced group, which in turn influenced their self-perceived competence. Gender should, therefore, not be assumed as a direct predictor of competence, as it was influenced by professional experience.

We also highlighted the region of pregraduate training as a significant factor in self-perceived competence. Physicians trained in the European Union (EU) had higher self-perceived competence scores compared to those trained in Switzerland. This finding may be attributed to differences in medical education, training programs, and physician-patient communication between regions [37,38,39] that could have influenced physicians’ self-perceived competence, as well as to potential contextual and cultural factors that can bear on self-assessment [36]. It is essential to recognize that this finding does not imply that one region’s training is superior to the other; rather, it highlights the influence of training background on self-perceived competence.

Previous formal training in BBN did not correlate significantly with self-perceived competence in delivering difficult news. This finding may seem counterintuitive, as one might expect that formal training in communication skills would enhance physicians’ confidence and competence in BBN [21,22]. However, it is important to consider several factors that could explain this absence of association. The effectiveness of formal training in BBN may depend on the nature, content, and quality of the training programs [22]. Less than half of the participants had taken part in BBN training and only a small minority at a pregraduate level. This was most likely due to the age structure of our sample and formal training in BBN having been generalized at a pregraduate level only over the last fifteen years [40]. Not all training programs are equally effective, and the specific communication skills and strategies taught may vary widely. It is likely that most training programs may not sufficiently address the unique challenges and emotional aspects of BBN in the context of childhood neurodisabilities. This study did not assess the specific content and quality of the formal training received by participants, which could explain the lack of association. It is possible that physicians place a higher value on experiential learning and on-the-job experience in developing their competence in BBN. While formal training can provide a foundation, practical experience and real-life interactions with patients and families may offer a more profound understanding of the complexities of BBN. The absence of a significant association with formal training may reflect the primacy of experiential learning in this domain. BBN in the context of childhood neurodisabilities is inherently complex, involving not only the communication of medical information but also addressing the emotional and psychological needs of parents and families [1,14]. Effective BBN requires a deep understanding of these nuances, which may be challenging to capture in formal training programs. This study did not assess whether the formal training programs specifically addressed the unique challenges of BBN in childhood neurodisabilities, which could be a critical factor. The lack of a significant association between formal training and self-perceived competence underscores the need for more tailored and comprehensive training programs in BBN, particularly in the context of childhood neurodisabilities. These programs should consider the unique challenges and emotional aspects of BBN and should provide opportunities for experiential learning and mentorship [41].

Physicians in this study identified several personal features and experiences as crucial for effective BBN. Empathy, preparation, adaptability, and personality were all considered important attributes. Importantly, they recognized the value of personal experiences, such as relationships with people with disabilities and parenthood, in enhancing their ability to deliver difficult news. This aligns with the broader literature on communication skills, which suggests that empathy and personal connection can greatly influence the quality of interactions with patients and their families [42,43].

The severity of the child’s diagnosis and the emotional reactions of parents significantly impacted physicians during BBN. This finding underscores the emotional challenges that healthcare professionals face when delivering bad news in the context of childhood neurodisabilities. Previous research has indicated that BBN discussions can evoke emotional responses in physicians and that the emotional impact can be a source of stress and burnout [12,28]. Therefore, recognizing and addressing the emotional toll of BBN is crucial for providing support to healthcare providers in this field.

The results of this study have practical implications for healthcare organizations and medical training programs. To enhance the competence of physicians in delivering difficult news, it is essential to provide training and support that addresses both the technical aspects of communication and the emotional resilience needed to navigate emotionally charged discussions. This support may include role-playing exercises, mentorship programs, and debriefing sessions [44] to help physicians not only process the emotional challenges associated with BBN but also engage in a reflexive process of continuous improvement throughout their careers. Physicians in our sample had little academic training in BBN (only two at a pregraduate level), whereas medical school training in BBN is now widely implemented. Our study did not explore whether the process of BBN was carried out according to international standards and the theoretical models of BBN, adhering to common frameworks such as the SPIKES, ABCDE, or BREAKS models [27]. There is emerging evidence that the impact of a BBN curriculum may increase if it reinforces previously learned skills, typically at a pregraduate level, and that additional reinforcement in the clinical setting could promote retention and improvement of competence in BBN [45]. This highlights the importance of not only theoretical knowledge but also concrete learning experiences, from as early as medical school and throughout physicians’ careers, to foster confidence and skills in BBN.

This study emphasizes the importance of building quality relationships with families and providing patient-centered care. In the context of childhood neurodisabilities, parents and caregivers often play a central role in a child’s care and decision making. Therefore, effective communication that considers the emotional needs of families is essential for ensuring that parents feel heard and supported during the BBN process [7]. Healthcare providers should be trained not only to convey information but also to provide emotional support and empathy, recognizing that the emotional well-being of parents is closely tied to the well-being of the child. Mnemonic tools such as NURSE (Naming, Understanding, Respecting, Supporting, Exploring) can prove useful in supporting empathetic communication and responding to emotion [46].

This study opens the door to further research in several areas. Comparative studies across different healthcare systems and cultures could provide insights into the cultural and contextual factors that influence BBN competence. As previous research has indicated a lack of clear correlation between self-perceived and actual competence in various fields of medical learning and practice [47], further studies should include assessments of both of these dimensions. Additionally, future research can evaluate the effectiveness of different training programs and interventions in improving physician competence and mitigating the emotional impact of BBN. These studies can help refine best practices and contribute to the development of evidence-based guidelines for BBN in the context of childhood neurodisabilities.

It is crucial to acknowledge the limitations of this study when interpreting its findings. First, this study’s reliance on self-reported data, including physicians’ self-perceived competence and attitudes, may introduce response bias. Physicians may tend to provide socially desirable responses, potentially inflating their perceived competence. Second, this study’s sample size is relatively small, and the participants were exclusively from Switzerland, which may limit the generalizability of the findings to broader international contexts. Additionally, this study’s cross-sectional design captures a snapshot of physician attitudes and competence at a specific point in time, but it does not provide insights into how these factors may evolve over time or in response to training interventions. Finally, this study primarily focused on physicians’ perspectives. Future research would strongly benefit from including the views of parents and caregivers to gain a more comprehensive understanding of the BBN process in childhood neurodisabilities. Despite these limitations, this study offers valuable insights into an underexplored area of healthcare and provides a foundation for future research and practice improvements.

In conclusion, this study highlights the intricate nature of BBN in childhood neurodisabilities and underscores the significance of physician competence and attitudes in delivering difficult news. It emphasizes the importance of professional experience and experiential learning and the significance of personal attributes and experiences, such as empathy, preparation, adaptability, and relationships with people with disabilities, in enhancing physicians’ confidence to deliver difficult news effectively. By addressing the practical and emotional challenges of BBN and providing appropriate training and support, healthcare providers can better support families and improve the overall experience of children with neurodisabilities and of their parents. Further research seems necessary, including comparative studies across different healthcare systems and cultures, longitudinal assessments of self-perceived and actual competence, and the development and exploration of training programs specifically geared towards BBN to parents of children with neurodisabilities. Despite its limitations, this study provides valuable insights into an underexplored area of healthcare, laying the groundwork for improvements in practice in a challenging and emotionally charged context.

## Figures and Tables

**Table 1:** Participant characteristics.

Characteristic	Number (*n*)	Percentage
**Specialty**		
Neuropediatrics	28	45.2
Developmental pediatrics	24	38.7
Pediatric rehabilitation	10	16.1
**Region of activity**		
German-speaking region	50	80.6
French-speaking region	12	19.4
**Region of pregraduate training**		
Switzerland	44	71.0
EU country	16	25.8
**Training in BBN**		
Pregraduate training	2	3.2
Postgraduate training	26	41.9

## Data Availability

The data presented in this study are available upon request from the corresponding author. The data are not publicly available due to to specific ethical and privacy considerations.

## Supplementary Materials

The following supporting information can be downloaded at: https://www.mdpi.com/article/10.3390/children10121854/s1, Table S1: questionnaire script.
