# Supplementary material for: Physicians’ Self-Perceived Competence on Breaking Bad News to Parents of Children with Neurodisabilities
Source: Children (Basel). 2023 Nov 26;10(12):1854. doi: 10.3390/children10121854 (PMC10741853; doi:10.3390/children10121854)
Supplement: Supplementary file 1 [file children-10-01854-s001.zip › children-2713000-supplementary.pdf]

Table S1: Questionnaire script (translated from the French version)

Physicians' practices and feelings when breaking bad news in neuropaediatrics, developmental paediatrics and paediatric neurorehabilitation.

The aim of this questionnaire is to explore your opinions, as experts of the field, on breaking bad news in paediatric neurodisability. The term "bad news" covers all announcements about neurological disabilities that have a significant impact on a child's life project.

1. What is your age? *N* years

2. What is your gender? *male, female, other*

3. What is your medical specialty?

paediatric rehabilitation, neuropaediatrics, developmental paediatrics, other (please specify)

4. How many years have you been practising in this speciality? *N* years

5. Where did you complete your pre-graduate medical training?

Switzerland, European Union, other (please specify)

6. Have you received specific training in breaking bad news? *yes, no*

7. If yes, at what level of training did you receive it?

pregraduate training, general paediatric training, subspeciality paediatric training, other (please specify)

8. Do you deliver bad news in your professional practice? *yes, no*

9. How many bad news announcements do you estimate you make per year? *N*

10. Do you feel competent when it comes to breaking bad news (1 = not at all competent, 10 = absolutely competent)? *N 1 – 10*

11. Generally speaking, doctors in your speciality are good at breaking bad news.

totally agree, agree, disagree, totally disagree

12. Breaking bad news is a skill that should be improved in your specialty.

totally agree, agree, disagree, totally disagree

13. Breaking bad news in your speciality is a skill that must be learned, and that is not innate.

totally agree, agree, disagree, totally disagree

13. Breaking bad news is a skill that can be developed through theoretical learning.

totally agree, agree, disagree, totally disagree

14. Professional experience is essential to breaking bad news in your speciality.

strongly agree, agree, disagree, strongly disagree

15. Personal experience is essential to breaking bad news in your speciality.

strongly agree, agree, disagree, strongly disagree

16. What makes personal experience essential is (1 = not important, 10 = very important):

Experience of parenthood *1 - 10*

Experience of bereavement *1 - 10*

Personal relations with people with disabilities *1 - 10*

Having received bad news as a relative *1 - 10*

Having already received bad news as a patient *1 - 10*

17. It is necessary to have a good relationship with the family to be able to break bad news properly.

totally agree, agree, disagree, totally disagree

18. How important are the following characteristics when breaking bad news (1 = not important, 10 = very important)?

Adaptability *1 - 10*

Empathy *1 - 10*

Preparation *1 - 10*

Physician's personality *1 - 10*

19. In your practice, it would be useful to organise debriefing sessions to discuss bad news announcements that may have been difficult, or how to improve bad news announcements.

strongly agree, agree, disagree, strongly disagree

20. What makes a bad news announcement emotionally impact you is more likely to be (1 = no impact, 10 = strong impact):

The severity of the diagnosis *1 - 10*

The child's age *1 - 10*

The parents' reaction *1 - 10*

21. What contributes to the severity of a disability in your speciality is (1 = not severe, 10 = very severe):

The impact on the child's quality of life *1 - 10*

The impact on the child’s life expectancy *1 - 10*

The impact on the child’s independence *1 - 10*

The lack of treatment *1 - 10*

The impact on the lives of their relatives *1 - 10*
